# A CD63 Homolog Specially Recruited to the Fungi-Contained Phagosomes Is Involved in the Cellular Immune Response of Oyster *Crassostrea gigas*

**DOI:** 10.3389/fimmu.2020.01379

**Published:** 2020-07-22

**Authors:** Conghui Liu, Chuanyan Yang, Mengqiang Wang, Shuai Jiang, Qilin Yi, Weilin Wang, Lingling Wang, Linsheng Song

**Affiliations:** ^1^Liaoning Key Laboratory of Marine Animal Immunology, Dalian Ocean University, Dalian, China; ^2^Key Laboratory of Experimental Marine Biology, Institute of Oceanology, Chinese Academy of Sciences, Qingdao, China; ^3^Liaoning Key Laboratory of Marine Animal Immunology and Disease Control, Dalian Ocean University, Dalian, China; ^4^Dalian Key Laboratory of Aquatic Animal Disease Prevention and Control, Dalian Ocean University, Dalian, China

**Keywords:** tetraspanin, phagosomes recruitment, receptor, innate immune response, *Crassostrea gigas*

## Abstract

Cluster of differentiation 63 (CD63), a four-transmembrane glycoprotein in the subfamily of tetraspanin, has been widely recognized as a gateway from the infection of foreign invaders to the immune defense of hosts. Its role in Pacific oyster *Crassostrea gigas* is, however, yet to be discovered. This work makes contributions by identifying *Cg*CD63H, a CD63 homolog with four transmembrane domains and one conservative CCG motif, and establishing its role as a receptor that participates in immune recognition and hemocyte phagocytosis. The presence of *Cg*CD63H messenger RNA (mRNA) in hepatopancreas, labial palps, gill, and hemocytes is confirmed. The expression level of mRNA in hemocytes is found significantly (*p* < 0.01) upregulated after the injection of *Vibrio splendidus*. *Cg*CD63H protein, typically distributed over the plasma membrane of oyster hemocytes, is recruited to the *Yarrowia lipolytica*-containing phagosomes after the stimulation of *Y. lipolytica*. The recombinant *Cg*CD63H protein expresses binding capacity to glucan (GLU), peptidoglycan (PGN), and lipopolysaccharide (LPS) in the presence of lyophilized hemolymph. The phagocytic rate of hemocytes toward *V. splendidus* and *Y. lipolytica* is significantly inhibited (*p* < 0.01) after incubation with anti-CgCD63H antibody. Our work further suggests that *Cg*CD63H functions as a receptor involved in the immune recognition and hemocyte phagocytosis against invading pathogen, which can be a marker candidate for the hemocyte typing in *C. gigas*.

## Introduction

Tetraspanins establish a conserved superfamily of four-transmembrane glycoproteins involved in a variety of cellular processes, such as cell development, proliferation, activation, adhesion, and motility (1–4). They are ubiquitous in various organisms, sharing a highly conserved architecture with the presence of four transmembrane domains, two extracellular loops containing one Cys–Cys–Gly (CCG) motif, and one intracellular tail containing cysteine palmitoylation sites (5, 6). Tetraspanins are depicted as a gateway for infection because of the roles in uptaking, trafficking, and spread of viruses as well as intracellular bacteria, fungi, and parasites (7). Extensively studied in mammals, 33 members of tetraspanins have been so far characterized and classified into four major subfamilies, namely, cluster of differentiation (CD), CD63, uroplakin (UPK), and retinal degeneration slow (RDS) subfamily (5–9). CD63, the first characterized tetraspanin, constitutes its own subfamily having a more ancient origin than the other tetraspanins (8, 10). CD63 and other tetraspanins interact with the receptors in the plasma membrane as well as themselves to build an interaction network termed as tetraspanin-enriched microdomains (TEMs) (11, 12). TEMs are always achieved by organizing multiple types of receptors and associated components (6, 7), which provide platforms to control pathogen binding, entry, and invasion, and subsequently the immune responses (13, 14). CD63 can interact directly or indirectly with various proteins, for example integrins, other tetraspanins, cell surface receptors, and kinases (15). CD63 regulates the trafficking of its interaction partners and mediates signal transduction events in the regulation of many membrane-associated processes (16). Growing evidences have demonstrated that tetraspanins also participate in the innate immune response, such as pattern recognition and signal transduction (17, 18). For example, CD63 and other tetraspanins such as CD37, CD9, and CD82 are involved in the pathogen recognition, pattern recognition receptor (PRR) complex formation, and antigen-presentation through their cooperation with other receptors (17, 19–21).

The characterization of CD63 has been recently found in invertebrates, for instance, disk abalone *Haliotis discus discus* (22) and clam *Paphia undulate* (23). Earlier work has observed an interesting phenomenon: under the stimulation with bacteria, virus, and pathogen-associated molecular patterns (PAMPs), the mRNA expressions of these CD63s change significantly. However, the detailed biological roles of CD63 are still not clear. So far, there are many reports on other tetraspanins in nematode, mollusk, arthropod, and ascidian (24–27), which could offer useful leads to the investigation of CD63 subfamily for invertebrates. For instance, invertebrate tetraspanins could be significantly induced by the stimulations of various pathogens (28–33), and they could function as mediators of innate immune response (34, 35). Tetraspanin D76 from insect (*Manduca sexta*) was found to play an important role in cell-mediated immune responses by influencing the encapsulation and clearance of bacteria (35). Tetraspanin from *Crassostrea ariakensis*, Ca-TSP, was also evidenced to associate with phagocytic bodies (25). Although increasing evidences point to the potential roles of invertebrate tetraspanins in innate immunity, most of them are largely limited to the gene diversity and mRNA expression profiles.

As invertebrate, the Pacific oysters (*Crassostrea gigas*) have been deemed as a model to investigate the innate immunity in mollusks (36, 37). The lack of specific cell markers, however, has greatly impeded the cell typing and innate immune research in invertebrates (38). This work investigates the functions of CD63 in Pacific oysters, aiming to offer new insights to understand the possible role of tetraspanins in the innate immune system of invertebrate, and provides a potential candidate marker for the cell typing. Specifically, a homolog of CD63 (designated *Cg*CD63H) is identified and characterized from *C. gigas*; the mRNA transcripts of *Cg*CD63H in different tissues as well as the temporal expression pattern after *V. splendidus* challenge are investigated; the binding ability of *Cg*CD63H to PAMPs is examined; and the roles of *Cg*CD63H during the phagocytosis process of hemocyte are revealed.

## Materials and Methods

### Oysters and Microbes

Adult oysters *C. gigas* (average shell length of 13.0 cm) were collected from a local farm in Qingdao, Shandong Province, China, and acclimatized in aerated fresh seawater at 15 ± 2°C for 10 days before processing. The oysters were fed with condensed microalgae, and the water was totally replaced daily.

Bacteria *Escherichia coli* Transetta (DE3) (Transgen), *Staphylococcus aureus* (Microbial Culture Collection Center, Beijing, China), *V. splendidus* (39), and fungi *Yarrowia lipolytica* (provided by Dr. Chi) were cultured in Luria–Bertani (LB) medium at 37°C, 2216E medium at 28°C, and Yeast Extract–Peptone–Dextrose (YPD) medium at 28°C, respectively. Then, the microorganisms were harvested and resuspended in sterilized seawater (SSW) and adjusted to the final concentration of 2 × 10^8^ CFU ml^−1^.

### Tissue Collection and Immune Challenge

Tissues including the hepatopancreas, mantle, gonad, labial palps, and gills were collected from six oysters as parallel samples. The hemolymphs were aseptically withdrawn from the posterior adductor muscle sinus of these six oysters by using a syringe and immediately centrifuged at 800 × g, 4°C for 10 min to harvest the hemocytes. All these samples were stored at −80°C after addition of 1 ml TRIzol reagent (TaKaRa) for RNA extraction.

For the bacteria challenge experiment, 200 oysters were randomly assigned into control, challenge, and blank groups. Eighty oysters individually received an injection of 100 μl sterilize seawater (SSW) were employed as the control group, while other 80 oysters that received an injection of 100 μl alive *V. splendidus* suspended in SSW (2 × 10^8^ CFU ml^−1^) were employed as the challenge group. These treated oysters were maintained in water tanks after injection, and 15 individuals were randomly sampled at 3, 6, 12, 24, and 48 h post-injection. The remaining 40 untreated oysters were employed as the blank group. Hemolymphs collected from three individuals were pooled into one sample, and there were five replicates for each sampling time point. The hemocytes were harvested and stored as described above.

### RNA Isolation and cDNA Synthesis

Total RNA was isolated from oyster tissues and hemocytes using Trizol reagent following its protocol (TaKaRa). The first-strand complementary DNA (cDNA) synthesis was carried out based on Promega M-MLV RT Usage information using the DNase I (Promega)-treated total RNA as template and oligo (dT)-adaptor as primer (Table 1). The reaction was performed at 42°C for 1 h, terminated by heating at 95°C for 5 min. The cDNA mix was diluted to 1:100 and stored at −80°C for subsequent gene cloning and SYBR Green fluorescent quantitative real-time PCR (qRT-PCR).

**Table 1:** Primers used in this study.

**Primer name**	**Sequence (5^**′**^-3^**′**^)**
**Clone primers**	
Oligo(dT)-adaptor	GGCCACGCGTCGACTAGTACT_17_
T7	GTAATACGACTCACTATAGGGC
*Cg*CD63H-F	ATGGGGTGTCGGGGTACC
*Cg*CD63H-R	AGTGAATGCGGTGGGTAAG
**RT-PCR primers**	
*Cg*EF1-α-rtF	AGTCACCAAGGCTGCACAGAAAG
*Cg*EF1-α-rtR	TCCGACGTATTTCTTTGCGATGT
*Cg*CD63H-rtF	GCTGGAATGCTGTGGAGGA
*Cg*CD63H-rtR	ACATCTGGCAGGTCTGGTAGT
**Recombination primes**	
*Cg*CD63H-exF	CGGGGTACCGGTGGAGTACGATGCCTTAG
*Cg*CD63H-exR	ATAAGAATGCGGCCGCAGTGAATGCGGTGGGTAAG

### The Cloning and Sequence Analysis of Full-Length cDNA

Sequence information of *Cg*CD63H (XM_011436987.3) was retrieved from the National Center for Biotechnology Information (NCBI) (http://www.ncbi.nlm.gov). A pair of gene-specific primers *Cg*CD63H-F and *Cg*CD63H-R (Table 1) was used for cloning of the full-length cDNA sequence. The PCR product was gel purified and cloned into the pMD19-T simple vector (TaKaRa). The resulting sequences were verified and subjected to cluster analysis. The phosphorylation, O-linked glycosylation, N-linked glycosylation, and methylation modifications of *Cg*CD63H were predicted by DISPHOS (http://www.dabi.temple.edu/disphos/), NetOGlyc 4.0 Server (http://www.cbs.dtu.dk/services/NetOGlyc/), NetNGlyc 1.0 Server (http://www.cbs.dtu.dk/services/NetNGlyc/), and GPS-MSP Online Service (http://msp.biocuckoo.org/online.php), respectively.

The tetraspanin homologs of *Cg*CD63H from some other species, including *Mus musculus, Xenopus tropicalis, Danio rerio, Salmo salar*, and *Oplegnathus fasciatus, Drosophila melanogaster, Tenebrio molitor, H. discus, Aplysia californica, Biomphalaria glabrata, Pomacea canaliculata*, and *Mizuhopecten yessoensis* were retrieved from NCBI (Supplementary Data 1). The domains of these proteins were predicted using the simple modular architecture research tool (SMART) version 7.0 (http://www.smart.embl-heidelberg.de/). Multiple sequence alignment of *Cg*CD63H and its homologs were performed with the ClustalW multiple alignment program (http://www.ebi.ac.uk/clustalw/). An unrooted phylogenetic tree was constructed based on the sequence alignment by the neighbor-joining (NJ) algorithm using the Mega 6.06 program (http://www.megasoftware.net/).

### The qRT-PCR Analysis

The qRT-PCR was carried out to investigate the mRNA expression of *Cg*CD63H. A fragment of 179 bp was amplified using two sequence-specific primers, *Cg*CD63H-rtF and *Cg*CD63H-rtR (Table 1), and the PCR products were sequenced to verify the PCR specificity. Two primers (Table 1) were used to amplify a 200-bp fragment of elongation factors (*Cg*EF1-α) as an internal control to verify the successful reverse transcription and calibrate the cDNA template. The SYBR Green qRT-PCR assay was carried out in an ABI PRISM 7500 Sequence Detection System (Applied Biosystems) as the previous description (40). All data were given in terms of relative mRNA expression using the 2^−ΔΔ*CT*^ method (41).

### Preparation of Recombinant Protein and Polyclonal Antibody of *Cg*CD63H

The cDNA fragment encoding the mature peptide of *Cg*CD63H was amplified with specific primers *Cg*CD63H-F and *Cg*CD63H-R (Table 1). A *Not*I site and a *Kpn*I site were added to the 5′ end of sense primer *Cg*CD63H-exF and antisense primer *Cg*CD63H-exR with the stop codon deletion, respectively. The PCR fragments were cloned into pMD19-T simple vector (TaKaRa), digested completely by restriction enzymes *Not*I and *Kpn*I (NEB), and then cloned into the *Not*I/*Kpn*I sites of expression vector pET-30a (Novagen).

The strain *E. coli* Transetta (DE3) with recombinant plasmid (pET-30a-CgCD63H) was incubated in LB medium (containing 75 μg ml^−1^ kanamycin), shaken at 220 rpm at 37°C. The control strain with plasmid pET-32a was incubated in the same medium with 100 μg mL^−1^ ampicillin. When the culture media reached OD_600_ of 0.5–0.7, the cells were incubated for an additional 4 h with the induction of isopropyl β-d-1-thiogalactopyranoside (IPTG) at the final concentration of 1 mmol L^−1^. The recombinant protein *Cg*CD63H (designated r*Cg*CD63H) and the recombinant protein Trx (designated rTrx) were purified by a Ni^2+^ chelating Sepharose column and refolded in gradient urea–Tris-buffered saline (TBS) glycerol buffer as the previous description (40). The resultant proteins were detected by sodium dodecyl sulfate–polyacrylamide gel electrophoresis (SDS-PAGE), and their concentration was quantified by bicinchoninic acid (BCA) kit (Beyotime).

For the preparation of polyclonal antibody anti-*Cg*CD63H, r*Cg*CD63H was injected into mice of 6 weeks of age to acquire polyclonal antibody as previously described (42). The serum from the identical mice before immunization was taken as negative control.

### Immunocytochemistry of *Cg*CD63H in Hemocytes

Hemolymphs were collected from the oysters cultured in filtered aerated seawater at 18°C for 1 week and immediately centrifuged at 800 × g, 4°C for 10 min to harvest the hemocytes. Modified Leibovitz L-15 media (Gibco) (43) were used to suspend the hemocytes. The hemocyte suspension was added into confocal dishes precoated with gelatin solution [gelatin, 5 g L^−1^; CrK (SO_4_)_2_·12H_2_O, 0.5 g L^−1^] and allowed them to adhere to the wall for 3 h. The supernatant was dislodged, and then, 4% paraformaldehyde (PFA; diluted in TBS) was added to fix the hemocytes for 15 min. After rinsing three times with TBST (TBS with 0.1% Tween-20), the dishes were blocked with 200 μl of 3% bovine serum albumin (BSA) [dissolved in phosphate-buffered saline (PBS)] at 37°C for 30 min. The supernatant was removed, and the dishes were incubated with 200 μl anti-*Cg*CD63H (diluted 1:1,000 in blocking buffer) as the primary antibody at 37°C for 1 h. After washing three times with PBST (PBS with 0.1% Tween-20), the dishes were incubated with Alexa Fluer 488-labeled goat-antimouse antibody (diluted 1:1,000 in blocking buffer) as the second antibody at 37°C for 1 h. After another three times of washing with PBST, 4′,6-diamidino-2-phenylindole (DAPI) (diluted 1:10,000 in PBS) was added into the dishes to stain the nucleus. After the last three times of washing, the dishes were mounted in buffered glycerin for observation with a laser scan confocal microscope (ZEISS).

### PAMP Binding Assay

The PAMP binding assay was performed according to previous report with modification (44). Briefly, 100 μL (20 mg) of lipopolysaccharides (LPS) from *E. coli* (Sigma-Aldrich, L2630-10MG), peptidoglycan (PGN) from *S. aureus* (Sigma-Aldrich, 77140-10MG), β-glucan (GLU) from *Saccharomyces cerevisiae* (Sigma-Aldrich, 346210-25MG), and mannose (MAN) (Sigma-Aldrich, M2069-25G) were adopted to envelop a 96-well microtiter plate (Costar). The wells were then blocked with 3% BSA (*w*/*v*) in PBS at 37°C for 1 h. After washed with PBS-T, 1/2-fold serial dilutions of r*Cg*CD63H in TBS (50 mmol L^−1^ Tris–HCl, 50 mmol L^−1^ NaCl, pH 7.6) were added in the presence of 0.1 mg mL^−1^ BSA or 1 mg lyophilized hemolymph. The same concentration of rTRX was used as negative control. After incubating at 18°C for 2 h followed by three times of washing, 100 μl mouse anti-His tag monoclonal antibody (Genscript, China) diluted to 1:2,000 was added and incubated at 37°C for 1 h. The plate was washed again, and 100 μl of rabbit-anti-mouse Ig-AP conjugate (Sangon Biotech, China) secondary antibody (diluted 1:2,000) was added and incubated at 37°C for 1 h. After the last washing, 100 μl of 0.1% (*w*/*v*) p-nitrophenyl phosphate (pNPP, Sigma) in 50 mmol L^−1^ carbonate bicarbonate buffer (pH 9.8) containing 0.5 mmol L^−1^ MgCl_2_ was added and incubated at room temperature in the dark for 30 min. The reaction was stopped by 2 mol L^−1^ NaOH, and the absorbance was measured at 405 nm. The wells with 100 μl of TBS were used as blank. The assay was repeated at least five times under similar procedures. Samples with *P*_sample_ – *B*_blank_/*N*_negative_ – *B*_blank_ (*P*/*N* value) > 2.1 were considered positive (44, 45).

### Phagocytosis Assay

Phagocytosis assay was performed according to previous report with modification (46). Microorganisms including *S. aureus, V. splendidus*, and *Y. lipolytica* were labeled with fluorescein isothiocyanate (FITC) to investigate the phagocytosis. All the microorganisms were grown to mid-log phase and harvested by centrifugation at 6,000 × g for 15 min. Cells were fixed with 4% PFA for 10 min, washed with 0.1 M NaHCO_3_ (pH 9.0) for three times, and then mixed with 1 mg mL^−1^ FITC (Sigma-Aldrich) in 0.1 M NaHCO_3_ (pH 9.0) buffer at room temperature with continuous gentle stirring overnight.

Briefly, hemolymph was collected from 15 oysters (5 in each group) and then centrifuged at 800 × g for 10 min to harvest hemocytes. The hemocytes were resuspended with 200 μl modified Leibovitz L-15 media. For hemocyte phagocytic rate assay, the hemocytes suspension was mixed with 20 μl of microbe culture (OD_600_ = 0.6, suspended in Tris–HCl) together with anti-CgCD63H and incubated in the dark for 1 h. The phagocytosis rate and phagocytosis index (mean FITC fluorescent intensity) were evaluated by FACScan flow cytometry (evaluated by BD, USA) following a previous research (47). PBS was used as blank control, and rTrx and negative serum were employed as negative controls. There were three replicates in each sampling. For the recruitment of *Cg*CD63H to *Y. lipolytica*-containing phagosomes assay, the hemocyte suspension was mixed with 20 μl of microbe culture (OD_600_ = 0.6, suspended in Tris–HCl) and incubated in the dark for 1 h. Endogenic *Cg*CD63H was detected by anti-*Cg*CD63H and visualized by DyLight 594-labeled secondary antibody (red). FITC-labeled *Y. lipolytica* (green) was selected to elicit the phagosome in oyster hemocytes. The nucleus stained by DAPI was observed in blue. The signals were investigated via fluorescence confocal microscopy based on previous description.

### Statistical Analysis

All data were given as means ± SE. The data were subjected to one-way analysis of variance (one-way ANOVA) followed by an unpaired, two-tailed *t*-test. Differences were considered significant at *p* < 0.05 and extremely significant at *p* < 0.01.

## Results

### The Molecular Characters and Phylogeny of *Cg*CD63H

**Figure 1:**
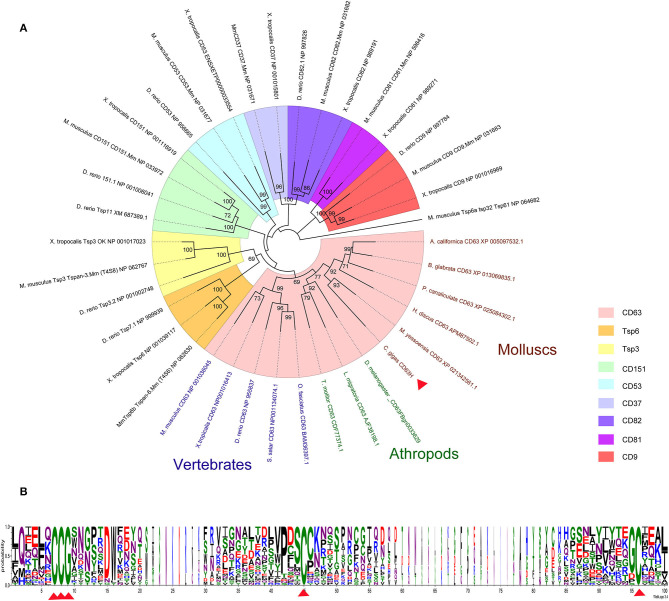
Phylogenetic analysis of *Cg*CD63H. The homologs of *Cg*CD63H from vertebrates (*Mus musculus, Xenopus tropicalis, Danio rerio, Salmo salar*, and *Oplegnathus fasciatus*) and invertebrates (*Drosophila melanogaster, Tenebrio molitor, Locusta migratoria, Haliotis discus discus, Aplysia californica, Biomphalaria glabrata, Pomacea canaliculata*, and *Mizuhopecten yessoensis*) were retrieved from the National Center for Biotechnology Information (Supplementary Data 1). **(A)** The unrooted tree was constructed by the neighbor-joining (NJ) algorithm using the Mega 6.06 program based on the multiple sequence alignment by ClustalW. The reliability of the branching was tested by bootstrap (1,000 replicates). Red arrow head indicated *Cg*CD63H. **(B)** The conserved residues and motifs in large extracellular loop were analyzed based on the multiple sequence alignment.

A cDNA fragment of 948 bp nucleotides representing the open reading frame (ORF) of *Cg*CD63H was amplified, which encoded a polypeptide of 315 amino acids with a molecular weight of 35.4 kDa and a theoretical isoelectric point of 5.37. There were four characteristic transmembrane (TM) domains, a short extracellular loop and a large extracellular loop identified in *Cg*CD63H (Supplementary Figures 1, 2). Additionally, conserved CCG motif and Cys residues were revealed in the large extracellular loop of *Cg*CD63H (Figure 1B). In bioinformatics prediction of post-translational modifications, five phosphorylation sites and six O-linked glycosylation sites were detected but no N-linked glycosylation and methylation site (Supplementary Figures 3–6).

On the basis of the multiple sequences alignment of tetraspanins, an unrooted phylogenetic tree was constructed by using the neighbor joining (NJ) method (Figure 1A). *Cg*CD63H and 37 homologs from other species were clustered into nine groups, including CD63, CD151, CD53, CD82, CD37, CD81, CD9, TSP3, and TSP6 groups (Figure 1A). *Cg*CD63H was successively bunched together with CD63s from mollusks and arthropods and then assigned into CD63 subgroup in vertebrates.

### The Tissue Distribution of *Cg*CD63H mRNA

**Figure 2:**
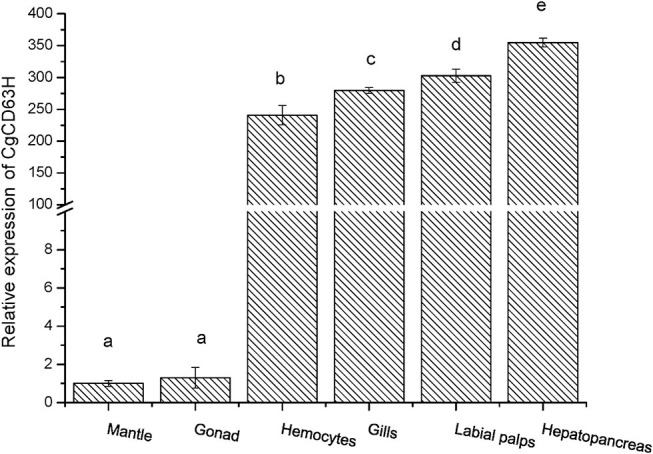
Tissue distribution of the *Cg*CD63H transcripts. *Cg*CD63H relative messenger RNA (mRNA) expression level in mantle, gonad, hemocytes, gills, labial palps, and hepatopancreas was normalized to that of *Cg*EF1-α. Each value was shown as mean ± SE (*N* = 6), and bars with different letters were significantly different (*p* < 0.01).

The qRT-PCR was employed to investigate the distribution of *Cg*CD63H mRNA transcripts in different tissues of oysters. A single peak was revealed in the dissociation curve analysis, indicating the amplification specificity for both *Cg*CD63H and *Cg*EF1-α (data not shown). The *Cg*CD63H mRNA transcripts were detected in all the tested tissues (Figure 2). A significantly higher *Cg*CD63H expression was observed in hepatopancreas, labial palps, gill, and hemocytes, which was 354.9-fold (*p* < 0.01), 302.7-fold (*p* < 0.01), 279.7-fold (*p* < 0.01), and 240.7-fold (*p* < 0.01) of that in mantle, respectively. The relative expression level of *Cg*CD63H mRNA in the gonad was 1.3-fold (*p* < 0.01) of that in mantle.

### The Temporal Expression of *Cg*CD63H mRNA in Hemocytes After *V. splendidus* Stimulation

**Figure 3:**
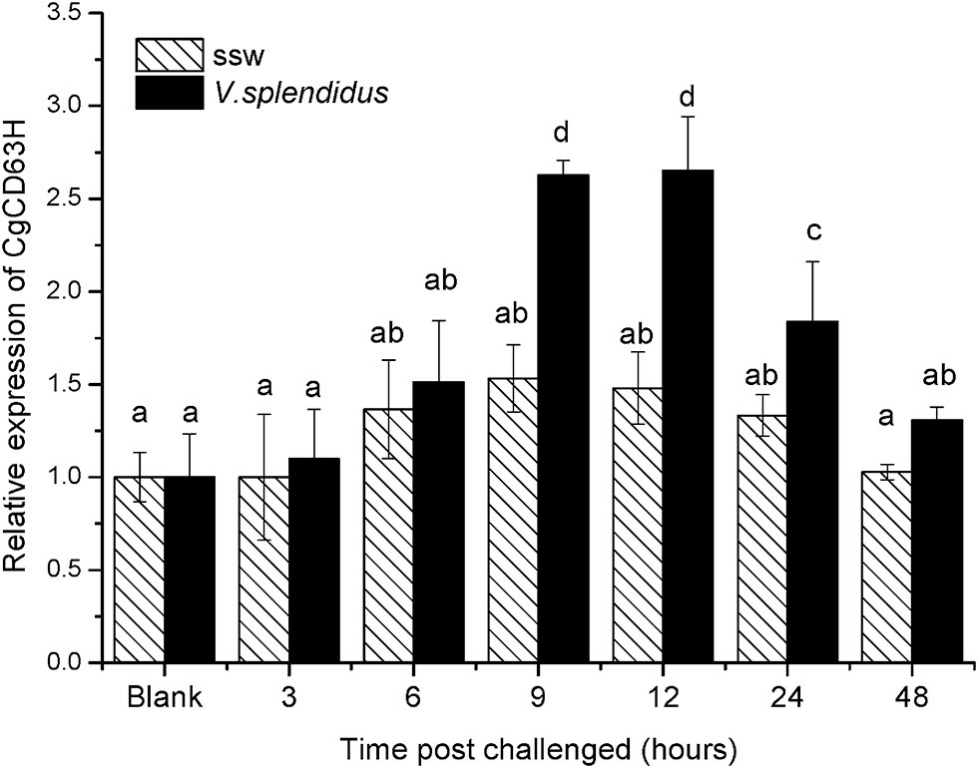
Temporal expression profile of *Cg*CD63H messenger RNA (mRNA) post-*V. splendidus* challenge. Oyster hemocytes were employed to analyze *Cg*CD63H expression, at 0, 3, 6, 12, 24, and 48 h after *V. splendidus* challenge. Comparison of the level of *Cg*CD63H mRNA (relative to *Cg*EF1-α) was normalized to 0 h, which was indicated as “relative expression level of *Cg*CD63H.” The values were shown as mean ± SE (*n* = 6), and bars with different letters were significantly different (*p* < 0.01).

The qRT-PCR was also used to detect the expression level of *Cg*CD63H mRNA in oyster hemocytes challenged by alive *V. splendidus* (Figure 3). The *Cg*CD63H transcripts were significantly upregulated at 9 h after *V. splendidus* challenge (1.71-fold compared to that in the control group, *p* < 0.01) and reached the maximum level (1.79-fold, *p* < 0.01) at 12 h, and then dropped back to the original level at 24 h (1.38-fold, *p* < 0.01). While no significant change on the expression level of *Cg*CD63H mRNA was observed in the control group during the experiment.

### The Recombinant Protein and Antibody of *Cg*CD63H

**Figure 4:**
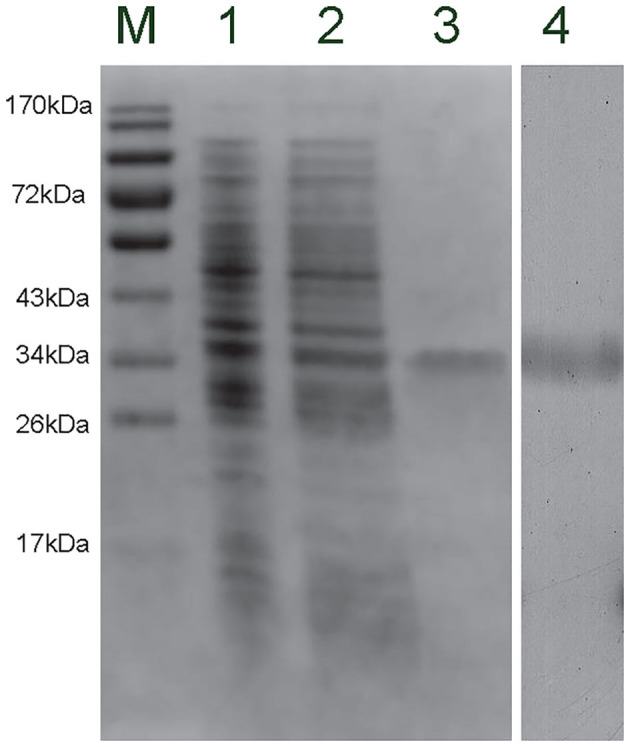
Sodium dodecyl sulfate–polyacrylamide gel electrophoresis (SDS-PAGE) and Western blot analysis of *Cg*CD63H. Lane M, protein molecular standard; lane 1, negative control for *Cg*CD63H (without induction); lane 2, induced r*Cg*CD63H; lane 3, purified r*Cg*CD63H; lane 4, Western blot using purified anti-*Cg*CD63H based on the hemocytes lysate sample.

The recombinant plasmid (pET-30a-*Cg*CD63H) was transformed into *E. coli* Transetta (DE3). After IPTG induction for 4 h, the whole cell lysate was analyzed by SDS-PAGE, and a distinct band with a molecular mass of 35 kDa was revealed (lane 3), which was in consistence with the predicted molecular mass of fusion recombinant protein of *Cg*CD63H with His-tag (Figure 4, lane 3). As control, no visible targeted band was detected in the group of cell lysate without IPTG induction. A polyclonal antibody against r*Cg*CD63H was prepared, and Western blotting analysis revealed a distinct single immune-precipitated band with a similar molecular weight predicted by the target sequence. This result suggested a high binding specificity of the polyclonal antibody against *Cg*CD63H (Figure 4, lane 4).

### Subcellular Localization of *Cg*CD63H in Oyster Hemocytes

**Figure 5:**
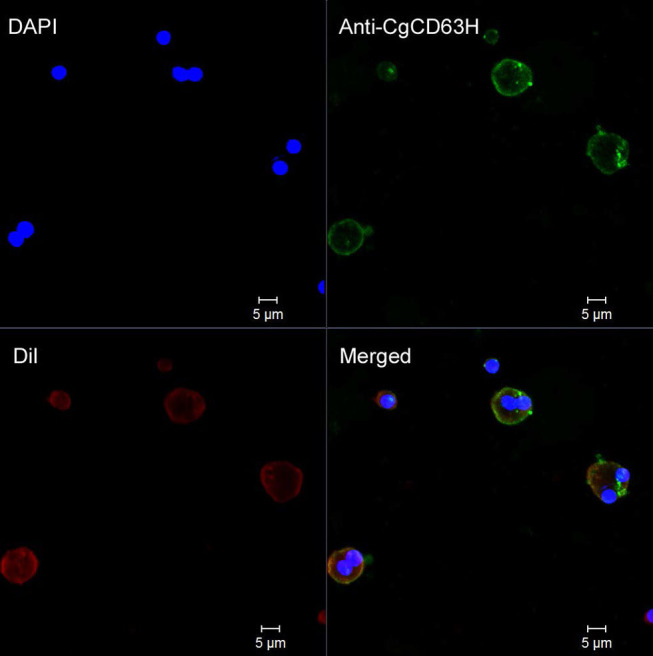
Subcellular localization of *Cg*CD63H protein in oyster hemocytes. Binding of antibody to *Cg*CD63H was visualized by Alexa 488-labeled secondary antibody (green); the nucleus of hemocytes was stained with DAPI (blue), bar = 5 μm.

Fluorescence confocal microscopy was employed to detect the localization of endogenous *Cg*CD63H in oyster hemocytes (Figure 5 and Supplementary Figure 7). The nucleus stained by DAPI was observed in blue, and the plasma membrane stained by DiI was observed in red. The *Cg*CD63H immunoreactive area was stained in green. The positive signal of CgCD63H appeared mainly over the plasma membrane of some oyster hemocytes (Figure 5 and Supplementary Figure 7).

### Binding Capacity of r*Cg*CD63H to Various PAMPs

**Figure 6:**
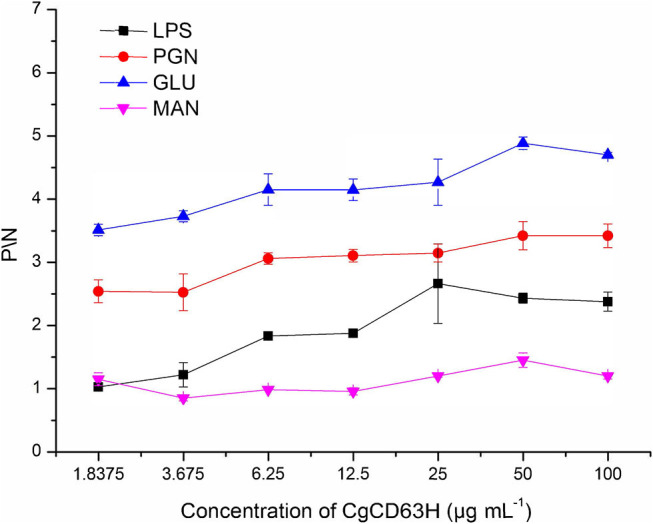
ELISA analysis of the interaction between *Cg*CD63H and the pathogen-associated molecular patterns (PAMPs). Plates were coated with LPS, PGN, GLU, and MAN, and then incubated with a series of concentrations of r*Cg*CD63H and rTrx at the presence of lyophilized hemolymph at 18°C for 2 h. After incubated with anti-*Cg*CD63H, the interaction was detected with goat antimouse Ig-alkaline phosphatase conjugate at 405 nm. Samples with *P*/*N* (*P*_sample_ – *B*_blank_/*N*_negative_ – *B*_blank_) > 2.1 were considered positive. Results are representative of the mean of three replicates ± SE.

The binding assay of r*Cg*CD63H to various PAMPs in the presence of lyophilized hemolymph was performed based on the OD_405_ value. The samples with *P*/*N* (*P*_sample_ – *B*_blank_/*N*_negative_ – *B*_blank_) > 2.1 were considered as positive (Figure 6). No positive value was detected in the absence of lyophilized hemolymph (data not shown). After the addition of lyophilized hemolymph, the *P*/*N* values of r*Cg*CD63H toward GLU, PGN, and LPS were all >2.1, under the minimum protein concentration of 1.8375, 1.8375, and 25 μg ml^−1^, respectively (Figure 6). r*Cg*CD63H possessed affinity to GLU, PGN, and LPS in a dose-dependent manner. The r*Cg*CD63H exhibited relatively higher affinity to GLU and PGN while lower affinity to LPS. The *P*/*N* values of r*Cg*CD63H for MAN were all <2.1 under the concentration of 1.8375 to 100 μg ml^−1^ (Figure 6).

### Recruitment of *Cg*CD63H to *Y. lipolytica*-Containing Phagosomes

**Figure 7:**
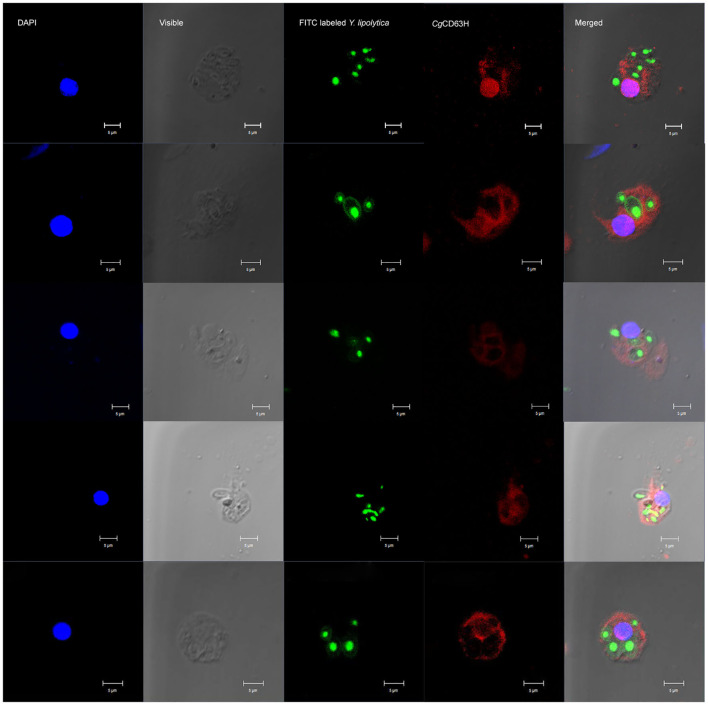
Specific recruitment of *Cg*CD63H to the *Y. lipolytica*-containing phagosomes. Fluorescein isothiocyanate (FITC)-labeled *Y. lipolytica* was selected to investigate the phagosome recruitment; the cell membrane and nucleus of *Y. lipolytica* were indicated with green signal. The hemocyte suspension was mixed with 20 μl of *Y. lipolytica* (OD_600_ = 0.6, suspended in Tris–HCl) and incubated in the dark for 1 h. Antibody anti-*Cg*CD63H was employed to detect the endogenic *Cg*CD63H. Binding of antibody to *Cg*CD63H was visualized by DyLight 594-labeled secondary antibody (red); the nucleus of hemocytes was stained with 4′,6-diamidino-2-phenylindole (DAPI) (blue), bar = 5 μm.

The recruitment of *Cg*CD63H after a phagocytic stimulus was investigated via fluorescence confocal microscopy (Figure 7). FITC-labeled *Y. lipolytica* (green) was selected to elicit the phagosome in oyster hemocytes. The nucleus stained by DAPI was observed in blue. Endogenic *Cg*CD63H was detected by anti-*Cg*CD63H and visualized by DyLight 594-labeled secondary antibody (red). In the absence of phagocytic stimulus, *Cg*CD63H-positive signal was detected on the surface of oyster hemocytes (Figure 5). After 1 h incubation with *Y. lipolytica, Cg*CD63H was highly enriched on the *Y. lipolytica*-containing phagosomes (Figure 7).

### The Change of Hemocyte Phagocytic Rate Post-anti-*Cg*CD63H Incubation

**Figure 8:**
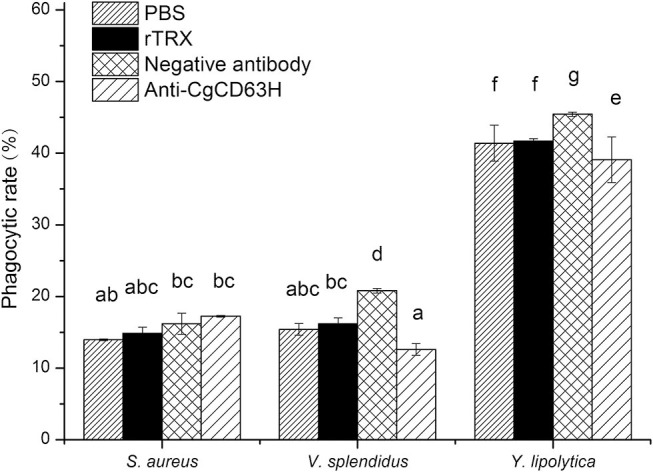
Hemocyte phagocytosis rate post-incubation of anti-*Cg*CD63H. Oyster hemocytes were employed to analyze the change in hemocytes' phagocytosis rate against *S. aureus, V. splendidus*, and *Y. lipolytica* post-incubation of anti-*Cg*CD63H. Fluorescein isothiocyanate (FITC)-labeled microbe, anti-*Cg*CD63H, and hemocytes were mixed and incubated for 1 h in dark. Recombinant protein rTRX and negative antibody were employed as negative controls. For each treatment, assay was performed in three different replicates for statistical analysis (*p* < 0.01).

Phagocytosis assay was performed on the basis of flow cytometry to test the phagocytic rate and phagocytic index of hemocytes after they were incubated with anti-*Cg*CD63H (Figure 8). The phagocytic rates of hemocyte toward Gram-negative bacteria *V. splendidus* and fungi *Y. lipolytica* were significantly down-regulated after they were incubated with anti-*Cg*CD63H, which were 0.60- and 0.86-fold (*p* < 0.01) compared to that of negative control group, respectively. However, in the Gram-positive bacteria *S. aureus* group, no significant change was observed after the hemocytes were incubated with anti-*Cg*CD63H compared to the negative control. The change in phagocytic index (mean FITC fluorescent intensity) indicated the same trend (Supplementary Figure 8). The phagocytic indices were 1.67 × 10^4^, 2.15 × 10^4^, and 4.69 × 10^4^ in the negative control group, and 1.78 × 10^4^, 1.30 × 10^4^, and 3.88 × 10^4^ in the anti-*Cg*CD63H blocking group for *S. aureus, V. splendidus*, and *Y. lipolytica*, respectively. Significant downregulation of phagocytic index after incubation with anti-*Cg*CD63H was detected in the *V. splendidus* and *Y. lipolytica* group, rather than in the *S. aureus* group.

## Discussion

CD63, as a member of tetraspanins, is an evolutionarily conserved protein family involved in multiple aspects of cellular physiological regulation (3, 4, 48). Although tetraspanins has been reported in a wide range of organisms, the functions of CD63 in invertebrate are, however, yet to be discovered (8). In the present study, a homolog of CD63 (designated *Cg*CD63H) was identified from genome database of oyster *C. gigas* with an open reading frame (ORF) of 948 bp encoding a polypeptide of 315 amino acids. The predicated amino acid sequence of *Cg*CD63H consists of four typical TM domains, a short extracellular loop, and a large extracellular loop. Additionally, a CCG motif and several Cys residues were identified in the large extracellular loop of *Cg*CD63H, which were highly conserved structural features of the previously reported CD63s (8, 22). Coincidentally, *Cg*CD63H was bunched together with CD63 homologs from invertebrates and vertebrates in an unrooted phylogenetic tree. These similar structure characters and reasonable phylogenetic relationships indicated that *Cg*CD63H was a member of the CD63 subfamily in mollusks, and it might function similarly to other CD63s.

CD63 has been reported to facilitate the immune response of various organisms (49). In the present study, CgCD63H was found to distribute in a wide range of tissues of *C. gigas* including the hepatopancreas, mantle, gonad, labial palps, gills, as well as hemocytes. The highest expression level of *Cg*CD63H was detected in the hepatopancreas, which was deemed as one of the most important tissues involved in the innate immune defense of mollusks (50). Labial palps, gills, as well as hemocytes were also deemed as important tissues involved in the innate immune defense of mollusks (51–53). The universal distribution of *Cg*CD63H in immune-related tissues led us to further investigate its roles in the immune response. The circulating hemocytes play indispensable roles in the immune response of mollusks against invading pathogens (51). *Cg*CD63H protein was typically distributed over the plasma membrane of oyster hemocytes. Moreover, the expression level of *Cg*CD63H mRNA in hemocytes increased significantly (*p* < 0.01) and reached the highest levels at 12 h after *V. splendidus* stimulation. In our previous publication, the transcriptome profiles of *C. gigas* in response to PAMP treatments were investigated by RNA-seq (54). Based on the published data (NCBI: SRR3110857), the temporal expressions of *Cg*CD63H were found to be significantly upregulated post-LPS stimulation (fold change = 2.07, *q* = 0), PGN (fold change = 1.74, *q* = 7.44E-16), and GLU (fold change = 1.57, *q* = 7.91E-07), indicating the potential role of *Cg*CD63H in the immune response against invading Gram-positive bacteria, Gram-negative bacteria, and fungi. The increased expression of CD63 in response to immune stimulation was depicted as its incipient character in immune response by multiple reports (25, 33). These results suggested that *Cg*CD63H could play important roles in immune defense of oysters against invading pathogens.

Immune recognition is the first step of immune response of mollusk which could discriminate non-self- from self-substances (55). Previous work has shown that mutual recognition between membrane receptors and pathogens leads to receptor clustering and membrane protrusions, which eventually enable microbial engulfment. Several mammalian tetraspanins including CD63 are reported to participate in the recognition of PAMPs through the binding activity with PRRs and tetraspanins traffic at the cell surface (11, 56). In invertebrates, CD63 is also suspected to function in recognition process with evidences of the upregulation of mRNA expression after PAMP stimulation (22). Here, *Cg*CD63H was found to bind GLU, PGN, and LPS only in the presence of lyophilized hemolymph, indicating that *Cg*CD63H could combine PAMPs in an indirect manner, possibly with the assistance of other possible PRRs. Earlier reports have demonstrated that CD63 and CD37 could interact with the C-type lectin Dectin-1, identified as fungi-PRR, through “tetraspanin web” and then initiate the antifungal immune responses *in vivo* (17, 57). A CD63 homolog from coleopteran beetle was also reported to possess the possible binding ability to PGN and GLU (49). The results supported that *Cg*CD63H could be involved in the regulation of immune recognition through the interaction of other PRRs within the “tetraspanin web,” for instance possible lectins or Ig domain containing cell adhesion molecules (IgCAMs). Since the recognition of the foreign invaders was the first step to initiate the immune response (58), the broad binding spectrum of *Cg*CD63H might endow it with a PRR partner role to organize PRRs on the cell membrane and induce the subsequent downstream immune responses against various intruders in oyster.

The previous reports have confirmed the effect of tetraspanins on phagocytosis in mammals (59–61). As a representative of tetraspanins, CD63 promotes the binding of outer segment particle through the interaction with specific PRRs instead of functioning as a direct receptor (57). In invertebrates, CD63 from calm *P. undulata* also plays roles in hemocyte-mediated phagocytosis (23). In the present study, *Cg*CD63H was recruited to the *Y. lipolytica*-containing phagosomes in the hemocytes of oysters after incubation with *Y. lipolytica*, which was similar to the previous observation in mammalian tetraspanins. The CD63 and CD82 were redistributed after immune stimulation and rapidly recruited to the membrane of nascent *C. neoformans*-containing phagosomes (19, 21). Additionally, in Figure 5, some positive signal of CgCD63H was found inside of the cell membrane, which might be caused by the phagocytosis of mixed debris in open circulatory system and resident microbiota, and the recruitment of *Cg*CD63H. Meanwhile, the phagocytosis of hemocytes against *V. splendidus* and *Y. lipolytica* was significantly inhibited after the incubation with anti-*Cg*CD63H. It was suspected that *Cg*CD63H promoted the phagocytosis through the interaction with multiple possible PRRs with its microdomains instead of acting as a direct invader binding receptor. In mammals, the interaction between tetraspanins and PRRs, such as CD81 and Toll-like receptor 4 (TLR-4) (62), was reported to be necessary in macrophage activation. Likewise, in invertebrates, the integrin–tetraspanin interaction was also observed in *M. sexta*, and the monoclonal antibody of tetraspanin CD76 could disturb the encapsulation of the invader, *Serratia marcescens* (35). An increasing body of evidence suggests that phagocytes play important roles in invertebrate innate immune responses. Cells of phagocytes were classified efficiently from the oyster *C. gigas*, which possess both potent oxidative killing and microbial disintegration capacities (63). Additionally, no significant change of phagocytic rate was found after the hemocytes were incubated with anti-*Cg*CD63H in the Gram-positive bacteria *S. aureus* group. It has been reported that PFAs have effects on some microbes (64). It was suspected that PFA might have impacts on *S. aureus* during FITC labeling, which influenced the immune response of oyster. Moreover, there might be other molecules involved in the phagocytosis toward *S. aureus*, and the phagocytic efficiency did not change significantly even though *Cg*CD63H was blocked by the antibody. As the phagocytosis of oyster hemocytes could be initiated by the recognition of invaders, the recruitment of *Cg*CD63H to the phagosome membrane further suggested the possible interaction between *Cg*CD63H and recognition receptors, as well as the partner roles of *Cg*CD63H in sensing the microbial content of phagosomes and eliciting an appropriate immune response. The difference in the PRRs interacting with CgCD63H may affect the specificity and efficiency in the immune response against different microbes. In the present study, *Cg*CD63H was found to enhance the phagocytosis of *V. splendidus* and *Y. lipolytic* other than *S. aureus*. These results indicated that *Cg*CD63H could interact with the PRRs against *V. splendidus* and *Y. lipolytic* with a higher affinity and promote a more efficient cellular immunity.

In conclusion, a member of the molluscan CD63 subfamily, *Cg*CD63H, was identified from *C. gigas*. *Cg*CD63H mRNA was mainly expressed in immune tissues and induced by the challenge of *V. splendidus*. The *Cg*CD63H exhibited binding activities to a wide spectrum of PAMPs in the presence of lyophilized hemolymph. Moreover, *Cg*CD63H was recruited to the *Y. lipolytica*-containing phagosomes of hemocytes after immune stimulation, and the phagocytosis of hemocytes was significantly inhibited after incubation with anti-*Cg*CD63H. All these results collectively indicated that *Cg*CD63H might function as a “gateway” between the pattern recognition of foreign invaders and the subsequent immune responses in oysters. CgCD63H could also be a promising marker in cell typing of phagocytic lines in *C. gigas*.

## Data Availability Statement

The raw data supporting the conclusions of this article will be made available by the authors, without undue reservation, to any qualified researcher.

## Ethics Statement

This animal study was reviewed and approved by Ethics Committee of the Institute of Oceanology, Chinese Academy of Sciences.

## Author Contributions

CL carried out cloning, expression, and purification of recombinant proteins as well as PAMP binding analyses. CY and SJ performed phagocytosis and flow cell cytometry analysis, while QY, WW, and MW carried out bioinformatics analyses. CL, LW, and LS designed the research and wrote the manuscript. All authors contributed to the article and approved the submitted version.

## Conflict of Interest

The authors declare that the research was conducted in the absence of any commercial or financial relationships that could be construed as a potential conflict of interest. The reviewer LZ declared a shared affiliation, with no collaboration, with several of the authors CL, MW, and SJ, to the handling editor at the time of the review.
