# Supplementary material for: A CD63 Homolog Specially Recruited to the Fungi-Contained Phagosomes Is Involved in the Cellular Immune Response of Oyster Crassostrea gigas
Source: Front Immunol. 2020 Jul 22;11:1379. doi: 10.3389/fimmu.2020.01379 (PMC7387653; doi:10.3389/fimmu.2020.01379)
Supplement: Supplementary file 1 [file Data_Sheet_1.PDF]

# A CD63 homologue specially recruited to the fungi-contained phagosomes is involved in the cellular immune response of oyster *Crassostrea gigas*

**Conghui Liu [1,2,3], Chuanyan Yang [1,3,4], Mengqiang Wang[2], Shuai Jiang[2], Qilin Yi [1,3,4], Weilin Wang [2,3,4], Lingling Wang [1,3,4*], Linsheng Song [1,3*]**

[1] Liaoning Key Laboratory of Marine Animal Immunology, Dalian Ocean University, Dalian 116023, China

[2] Key Laboratory of Experimental Marine Biology, Institute of Oceanology, Chinese Academy of Sciences, Qingdao 266071, China

[3] Liaoning Key Laboratory of Marine Animal Immunology and Disease Control, Dalian Ocean University, Dalian 116023, China

[4] Dalian Key Laboratory of Aquatic Animal Disease Prevention and Control, Dalian Ocean University, Dalian 116023, China

*** Correspondence:**

Prof. Linsheng Song, Lingling Wang

lshsong@dlou.edu.cn, wanglingling@dlou.edu.cn

**Keywords:** Tetraspanin, Phagosomes recruitment, Receptor, Innate immune response, *Crassostrea gigas*

# *Supplementary Material*

## 1 Supplementary Data

**Supplementary Data 1.** The homologs of cgCD63 employed in phylogenetic analysis

Mouse

>Mm.CD151_CD151.Mm__NP_033972

MGEFNEKKATCGTVCLKYLLFTYNCCFWLAGLAVMAVGIWTLALKSDYISLLASSTYLATA
YILVVAGVVVMVTGVLGCCATFKERRNLLRLYFILLLIIFLLEIIAGILAYVYYQQLNTELKEN
LKDTMVKRYHQSGHEGVSSAVDKLQQEFHCCGSNNSQDWQDSEWIRSGEADSRVVPDSCC
KTMVAGCGKRDHASNIYKVEGGCITKLETFIQEHLRVIGAVGIGIACVQVFGMIFTCCLYRSL
KLEHY

>Mm.CD53_CD53.Mm____NP_031677

MGMSSLKLLKYVLFIFNLLFWVCGCCILGFGIYFLVQNTYGVLFRNLPFLTLGNILVIVGSIIM
VVAFLGCMGSIKENKCLLMSFFVLLLIILLAEVTIAILLFVYEQKLNTLVAEGLNDSIQHYHSD
NSTMKAWDFIQTQLQCCGVNGSSDWTSGPPSSCPSGADVQGCYNKAKSWFHSNFLYIGIITI
CVCVIQVLGMSFALTLNCQIDKTSQALGL

>Mm.CD9_CD9.Mm___NP_031683

MPVKGGSKCIKYLLFGFNFIFWLAGIAVLAIGLWLRFDSQTKSIFEQENNHSSFYTGVYILIGA
GALMMLVGFLGCCGAVQESQCMLGLFFGFLLVIFAIEIAAAVWGYTHKDEVIKELQEFYKD
TYQKLRSKDEPQRETLKAIHMALDCCGIAGPLEQFISDTCPKKQLLESFQVKPCPEAISEVFN
NKFHIIGAVGIGIAVVMIFGMIFSMILCCAIRRSREMV

>Mm.CD81_CD81.Mm___NP_598416

MGVEGCTKCIKYLLFVFNFVFWLAGGVILGVALWLRHDPQTTSLLYLELGNKPAPNTFYVG
IYILIAVGAVMMFVGFLGCYGAIQESQCLLGTFFTCLVILFACEVAAGIWGFVNKDQIAKDV
KQFYDQALQQAVMDDDANNAKAVVKTFHETLNCCGSNALTTLTTTILRNSLCPSGGNILTP
LLQQDCHQKIDELFSGKLYLIGIAAIVVAVIMIFEMILSMVLCCGIRNSSVY

>Mm.CD82_CD82.Mm__NP_031682

MGAGCVKVTKYFLFLFNLLFFILGAVILGFGVWILADKNSFISVLQTSSSSLQVGAYVFIGVG
AITIVMGFLGCIGAVNEVRCLLGLYFVFLLLILIAQVTVGVLFYFNADKLKKEMGNTVMDIIR
NYTANATSSREEAWDYVQAQVKCCGWVSHYNWTENEELMGFTKTTYPCSCEKIKEEDNQL
IVKKGFCEADNSTVSENNPEDWPVNTEGCMEKAQAWLQENFGILLGVCAGVAVIELLGLFL
SICLCRYIHSEDYSKVPKY

>Mm.CD63_CD63.Mm__NP_001036045

MAVEGGMKCVKFLLYVLLLAFCACAVGLIAIGVAVQVVLKQAITHETTAGSLLPVVIIAVG
AFLFLVAFVGCCGACKENYCLMITFAIFLSLIMLVEVAVAIAGYVFRDQVKSEFNKSFQQQM
QNYLKDNKTATILDKLQKENNCCGASNYTDWENIPGMAKDRVPDSCCINITVGCGNDFKES
TIHTQGCVETIAIWLRKNILLVAAAALGIAFVEVLGIIFSCCLVKSIRSGYEVM

>MmCD37_CD37.Mm__NP_031671

MSAQESCLSLIKYFLFVFNLFFFVLGGLIFCFGTWILIDKTSFVSFVGLSFVPLQTWSKVLAVS
GVLTMALALLGCVGALKELRCLLGLYFGMLLLLFATQITLGILISTQRVRLERRVQELVLRTI
QSYRTNPDETAAEESWDYAQFQLRCCGWQSPRDWNKAQMLKANESEEPFVPCSCYNSTAT
NDSTVFDKLFFSQLSRLGPRAKLRQTADICALPAKAHIYREGCAQSLQKWLHNNIISIVGICL
GVGLLELGFMTLSIFLCRNLDHVYDRLARYR

>Mm.Tsp6a__tsp32_Tsp61__NP_064682

MGHWNRIKIAKCQILITNFLVLLLGLSMATMVVVIHFGDHFTVIGHASLERNPYETLRYWAF
YVGISLAGLLSLGAALSTIATVREAHGLMAAGFLCFALSFCILVQVAFWRFYNPTQVEDAVL
DTYDFVYDQAMKSPSSNWWQELAVIQDTFLCCGKKSPFGLLVSTGAIMCQGREAMREDCL
QSIRNVLWTHYSIASILTCTSLALTVYAMMLCAFLWFAIHSYHGLDRKGRYSLTPPRSHGFQ
TQEPSLFRWT

Frog

>Xt.CD53_ENSXETP00000033554

MASNFVNTLKYLMFAFNFLFWVTGCSIIAIGIYFVVNNIYGDLLTNNPSLTVGNALIAIGIIIM
VFGFLGCMGAIKENKCLLLTFFILLLLILLAEVIMAILLFVYEKQLDNYVRDRLTSSFEQNLKQ
NSSETWNIIQRNLQCCGINGTKDWKDNIPNSCCASNANSKCSQADLFKMGCSEALKNWFEK
NFLYVGVGTICISVIEVLGMSFALTLYCHISRSSGTLST

>Xt.CD9_NP_001016989

MPVKGGIKCIKYLLFAFNFIFWLAGTAVLGIGLWLRFDPQTKAMFEADQNTGAFYTGVYILI
GAGALMMLVGFLGCCGAIQESECMLGLFFAFLLVIFAVEIAAGIWGFTNKDKVVDELKTFY
KDTYTKYMKSNDNALKDTLKAIHLALNCCGMTGVLEATVTDICPNNQGIVGALSAQSCPAA
IEDVFTTKFHIVGAVGIGIAVIMIFGMIFSMVLCCAIRNNREMV

>Xt.CD81_NP_989271

MGVEGCTKCIKYLLFVFNFIFWLAGGVILGVALWLRHDPQTSNLLFQQFEDKHAPGTFYIGV
YIIIAVGAVMMFVGFLGCYGAIQESQCLLGTFFACLVILFACEVAAGIWGFVNRDQVSKEMR
LFYSEVYQHATTGTKEQQQKALPVLKAFHETLQCCGDASLKKYVTMSITDMCPKRSNILEQI
TIEDCHQKIDVLFSTKLYLVGIAAVVVAVIMIIEMIFSMVLCCGIRIYSVY

>Xt.CD82_NP_989191

MATSGCMKVTKYFLFLFNLLFFILGAVILGFGIWILVDKTSFIAVLQTSSSYLRTGSYILIAVG
GFTMVMGFLGCLGAVNEIRCLLGLYFSFLLIILIAQVAAGILIYLQRDTLKTEMSSIIHELIVTY
DYEDGKNTSAETTWDYIQTNIRCCGWTNYQNWTANTVVQNSTNQYPCSCMKNQSSPNGFC
QYNGTVESAVYQTGCMQGVESWLQDNLGIILGVCVGVAVIELLGLILSMCLCRSIQSEDYTK

VPKY

>Xt.CD37__NP_001015801

MAIKGCLSVTKYFLFVFNLLFFILGGVLLCFGLWILFDRGSFATMIGSSVPTLKVWSYVFSGV
GILTMLLGFLGCLGSLKEIKCLLGFYFAFLLLLFSAQITIGVLVYTQRNSLSTRLGTIVESVIKD
YGLYANQTDLEEGWDFAQEKMQCCGWYTPDDWMKNQKIQNTSVSLYPCSCRNVSAFHTN
SSANSSTEFPEAKAGFCTTGAREKWPVYQTGCMNTLQAFLLNNSITIVGVCIGIALLELFVMT
LSMCLCRNLDQNYNKLARYS

>Xt.CD63__NP_001016413

MAVEGGMKCVKFLMFFFNFVFWVCGIALIAIGIYVQIQLNHTLIMKNATSSGAPIVIIVVGVV
IFLIAFFGCCGALKENYCMVTTFAVVLVLIFLVEIAAAIAAYVYKDKLRTAFEDSFKNGMVH
YNDTKDIADSIDLLQKEFQCCGAINSTDWRQYAPFTGTNTVPDSCCKNITAGCGKGPIANINS
EGCATGIDQWVKKNVGIVAGVALGIALFEILGIVFACCLMKGIRSGYEVM

>Xt.CD151__NP_001116919

MSEYNEKKETCGTICLKYLLFTFNFFFWLAGLAVMAVGIWTLIQKSDYISLLPSNTYAATAYI
LVIAGAIVMITGILGCCATFKERKSLLKVYFILLLCIFILEVLAGILAYIYYQQLNAELKQSLKQ
TMTTKYKQPGEEKVTNAVDKLQQEFKCCGSNNSEDWRDSIWINSPEAEKRLVPDSCCKTVT
QRCGIRDHPSNIYKTEGGCITKLETFIRAHLLIIGAVGIGIACVQLFGMIFTCCLYRSLKSEPY

Zebrafeish

>Dr.CD53___NP_956665

MSCLKCLKYIMCVVNFIFFICGAAIFGMGIYLMTFSRLSLLPSLQAMSIANTLFITGIIITCVSFL
GFLGALKENRCLLISFFILLFILMLAELAAACLMLMYESKIENFIKDDLVDGLNQSIKNRKQH
NTTDDWDKVQETFGCCGIQNATDWQGFVPQSCNISGTSNWHKGCFKLLENSFESNLLSTGI
GVIVVCIIEVLGMCFSMTLFCHINRSGLGYK

>Dr.CD151.1__NP_001006041

MADAEEKTNSCGTVCLKYLLFVFNLLFWLAGGAVMAVGIWTLLDKSDYISLLSSNTYMVA
AFILIGAGAVVVFTGILGCCATIREQRSLLIVFLILLLLIFLLEITAGVLAYVYYQELNAELRAD
LKERMVENYQQPGQEHITRAIDNLQQDLKCCGSNSSADWRDGAWIRNYADRRLVPDSCCK
TPSVGCGVRDHPSNIYKVEGGCISKLEEFILQHLLILGSVGLGIAFVQIVGMIFTCCLYRSLKEE
IY

>Dr.Tsp11_XM_687389.1

MGSMYKDEQGDWITVCLKYLLFVFNFLFWMGGGVVMGVGIWTLVDKGEYLSLLASSTFA
VSAYILILAGGLVMVTGFLGCCAVIREQRSCLSTYFSCLLLIFLIELVAGVLAYVYYQALSEEL
KQHLSKTMMENYAQPGKESITQSVDRLQQDFKCCGSNNSLDWMHSVYIMSQAADSRVVPD
SCCKTITPQCGRRDHPSNIYKVEGGCITKLEQFLADHLLIIGAVGIGVACLQICGMVFTCCLHR
RIKLDPY

>Dr.CD9___NP_997784

MGVEGCPKCIKYSMFLLNSVFWIAGTAVLAVGLWLRFDPKTKSLFEGENSPYVFYTGVYILI
AAGALMMVVGFFGCCGAIQESPCMLGLFFFFLLVIFAVEVAAGIWGFSNQTKVTEDITTFYR
QTYDTYQQSKQEALKKTLRLFQHGLNCCGPSGNMQESLDETCPKKEGLDNLIIKSCPDAIDE
VFNSKLHIIGGVGIATGVIMIFGMIFSMMLCCAIRKTREIV

>Dr.CD82.1___NP_997826

MGKGCITATKYFLFLFNFIFFIFGATIMGFGLWILLDNQSLIAVLQESSVILKVVSYILIGVGSFS
MLLGFLGCLGAIYEIRCLLGLYFTCLLLILLAQVAVSILIYFQRDLLKTEADKIVSQVVANYPG
QNKTAEQAWDYLQRTMQCCGWNGRMDWDENHIIKNNTVPLYPCSCHNYSIQAPIVPDNGF
CQASSSDWPIYQTGCLEHVGSWLFTNYGIILGICLGVAVIELLGMIFSMGLCKSVHQEDYTK
VPKY

>Dr.CD63__NP_955837

MAVEGGAKCVKYLLFFFNFIFWLCGLALIVLGILVHVSLHNTAILQGASGSPMVLIVVGVIIF
FISFFGCCGAWKENQCMVVTFAIILSLIVITEIGAGIAGYIFRGKVNELLDQSFNTMIAGYNKT
EEYRTTLDSIQKQLKCCGGNSSSDWVNFSADHISVPDSCCKNVTKNCGIGAMTKPTVIYLEG
CQPILETRIKENILWIAVGALVIGFVQITGIVLACILSRAIRSGYEVM

Other vertebrates

>NP_001134074.1 CD63 antigen Salmo salar

MGVEGGMKCVKYLLFFFNFIFWLCGLALIVLGVLVQVALHNTVVINNVSASSAPIVLIVVGV
VVFFIAFFGCCGAWKESYCMVTLFSILLGLIIITEIGAAIAGYVFRGNLTVIVHESLNDMVTKY
SNGTDEFQKVLDNLQIDLKCCGVINATDWRGNFGSGTNSVPDSCCVNVTAGCGQGAMEDS
SKVHQMGCQTVVEELLKKNMMWVIVAALVIAFLQIMGIIFACMLMRGIRSGYEVV

>BAM36397.1 CD63 antigen Oplegnathus fasciatus

MGVAGGMKCVKFLLFFFNFIFWLCGLALIVVGILAQVALHNSFMISDPSASGAPIVLIGVGVV
IFFIAFFGCCGAWKENYCMVTTFAVLLSLIIIIEIAAAITGYVFRSKLSTVVQDSLTDMIVNYK
NSTDKFRETVDKLQEDLKCCGVNSSSDWRHFKPDGDSVPDSCCVNVSTNCGVRAMTDAAK
VYQKGCHDVVEALLKKNIQWVIVAALVIAFLQIMGIVFACVLMRGIRSGYEVM

Athropoda

>FBgn0033629_CD63_ Drosophila melanogaster

MRSCGPSLIKYVLFAFNVLFAISGLGILIAGAVVLADVNEFNHFVEGRVLAPPIVLIVTGLIIFLI
ASLG CFGAIKESPT LLITFAVLLAVIFIVELAVGIAASVFKKDLEGMVKNSLQESIKRSNSEDT
MAWDNIQQKL MCCGVDSPADWRTLSANKTLPGSCCQPQYIDSTVGHCLESPALGKDKYFQ
VGCVGKLKDR IEKNAIILIG VGIGIAFIQI LGIVLACYLA NSIRQERAK

>AJF38198.1 cd63 Locusta migratoria

MVSGGMSCVKYLLFTFNLLFVISGIVIVSVGALALSNFSSYDPFFGGTVIAGPIVLLIVGVIVFI
VAFFGCCGALKENHCMVVTFSVLLLIIFAMELGGGIAGYVLKEVLKDDVDNQLNSTLSKYG
SNEDITKSWDIMQTDLHCCGINGPDDWIKAGMKIPASCCSSGNHVNEVCGSPPFTNGCVESL
ENFITHRAVVLGGVGIGIAFVQLVGVILACCLAKSIRKEYETV

>CDF77374.1 CD63 protein Tenebrio molitor

MVSGGMTCVKYLLFVFNLLFFVSGVVMLAIGAVIYVVYAHYYNFVYDSFQSAPLILIIVGVII
FLVAFFGCCGACKENHCMIITFSVLLLVIFTLEVGTGIAGYIRRNEVETMLENKLNSTMFDYY
NNEKIKETWDIAQHEAKCCGMNGPDDWRRVIHNDTLPHTCCPDTPDDGSCTNKSPNVYKD
ACFFKLKEVFTKYGSIIGGVGIGIAICQLMGVIFACCLARSIRQEYETV

Mollusca

>APM87502.1 CD63 Haliotis discus discus

MEGGMKIVKGLLMVFNIIFVIVGCALIGVGAYVQTQLTDVASIFGSEYNGPGILLIFVGVIIFLI
AAFGCLGACRENHCCIMTFAGLLVVIFILEIGGGIAGFVLRDQIESTAVKKMEEAQGKFNKTD
VKEGWKFIQQSFKCCGADNYTSWEQFLPQPPESCCKSIGSIECKDRSYNKTDGIYTKSCTKGI
IDWLKSNVILLGGIGIGIGLAFVQVFGICLACCLGKAIKKEYEVV

>XP_005097532.1 PREDICTED: CD63 antigenlike Aplysia californica

MVEGGMKCIKILLFVFNLLFVIAGIGLIATGAYVNIKMDEYYDFFGKEYLGPGILVIIVGVLIF
LIAFFGCCGAIRENYCLTMTFAVSLAIIFILEIAGGITGFVLRDKIEDDVKGVLTDAMRNYNES
HHEGVTKSWNKLQEEFSCCGVNNYTDWNTTRGLSAPPVSCCAKIPCTPSFDDPSSIYTDPCA
GQIEDWLKGKVAIIGGVGIGLAFVQVVGVMFACCLARAIRKEYEVV

>XP_013069835.1 PREDICTED: CD63 antigenlike Biomphalaria glabrata

MVEGGMKCVKYLLFVFNLIFVIAGIGLIAAGAYVKVKLDQYYDFFGSDYVGPGILLIIVGVII
FLLAFFGCCGAIKENYCLTMTFAVFLGIIFVLEIAAGIAGFVLRDDIDREIDDILTKTLPKYNNS
SGIRKTWDSLQDEFHCCGPDNYTQWKSMGIFSGGNLPASCCKDSKLACSTNDTSNIYNEGC
VSKFEDWLKDKVAIIGGVGIGLAFVQVVGILFACCLARAIKKEYEVV

>XP_025084302.1 CD63 antigenlike Pomacea canaliculata

MVEGGMKCIKFLLFAFNLIFVIAAIAIIAVGAYVQIKLTEYYDFFGNQYAGPGILLIVVGVFIFI
IAFFGCMGAIKENYCLVMTFAVLLALIFILMIAGGIAGFVLRNDIEDKVVDVLKNEVTNYNK
NPGVTDAWNVLQQEFQCCGVTNSSDWKQASDLNTTYPWSCCMDSEACKTKQNITYDDIYK
QACESAFIGWMKDKVAIIGGVGIGLAFVMVVGVLFACCLARAIRKEYEVV

>XP_021342561.1 CD63 antigenlike Mizuhopecten yessoensis

MVEGGMKCVKLLLFAFNFIFVVVGCVLIGVGTYIQVQFKDYLNFVGQFSSGPGALLIAVGVII
FFIAAFGCCGAWKENYCLVMIFAVLLGIVFILMIAGGIAGYVLHDKVEDGVKTLMNDSMKR
YDADPTIWDLVQKDFKCCGTNSSEDWKNVNNGTVPASCCPPDGTCDGKGSGVYSVGCLDA
FVDWVKDKIFIVGGVGIGLAFVQIVGILFACCFGRALKKEYEVV

## 2 Supplementary Figures

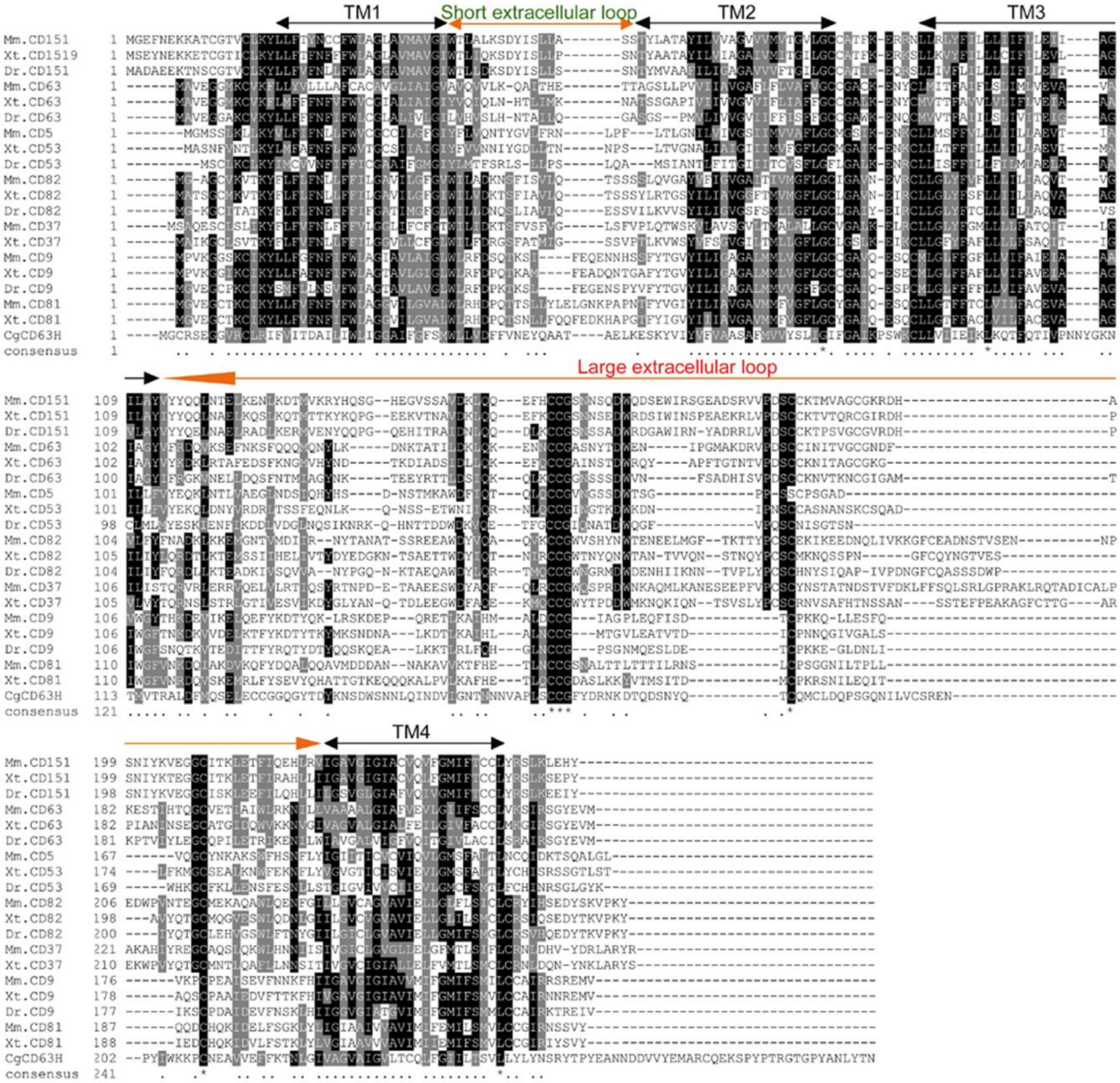


**Supplementary Figure 1.** Multiple sequence alignment of the tetraspanins by ClustalW2. The tetraspanin homologs of CgCD63H from Mus musculus, Xenopus tropicalis and Danio rerio were employed for multiple sequence alignment and sequence information was described in supplementary 1. Amino acid residues that are conserved in at least 60% sequences are shaded in dark, and similar amino acids are shaded in gray. Asterisks below indicate the CCG motif and Cys residues that are conserved throughout the tetraspanins. TM domains (TM1, TM2, TM3, and TM4), short extracellular loop and long extracellular loop were indicated in arrows.

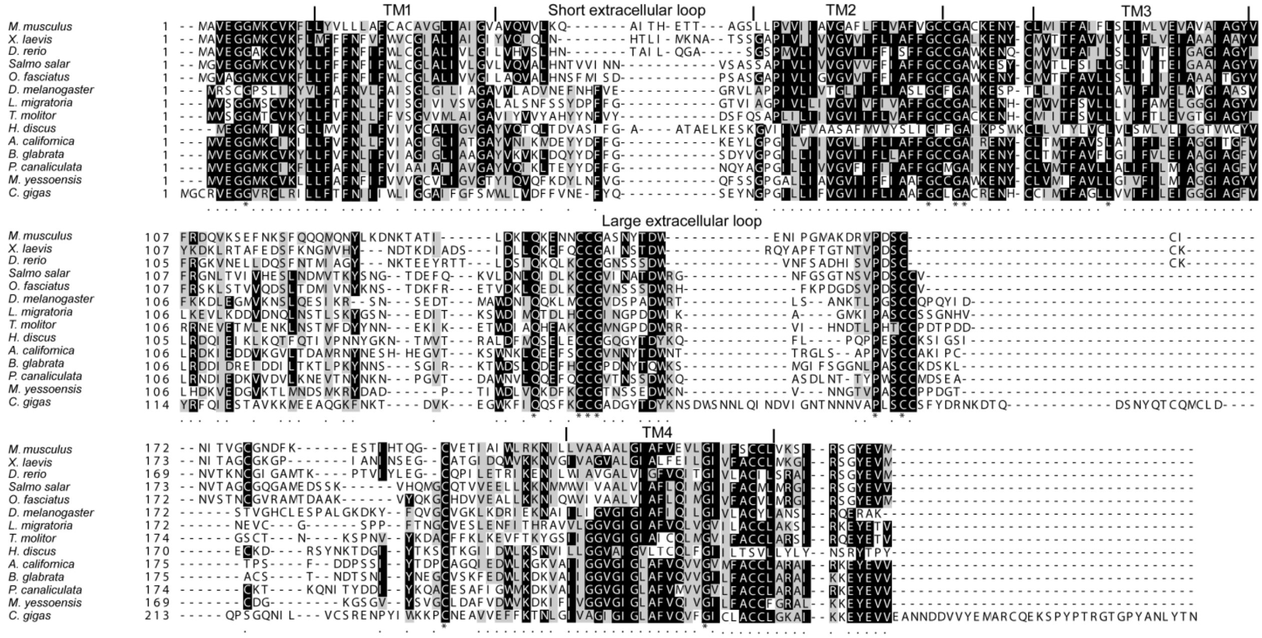


**Supplementary Figure 2.** Multiple sequence alignment of the CD63 homologs by ClustalW2. The homologs of CgCD63H were employed for multiple sequence alignment and sequence information was described in supplementary data 1. Amino acid residues that are conserved in at least 60% sequences are shaded in dark, and similar amino acids are shaded in gray. Asterisks below indicate the CCG motif and Cys residues that are conserved throughout the tetraspanins. TM domains (TM1, TM2, TM3, and TM4), short extracellular loop and long extracellular loop were indicated upon the alignment.

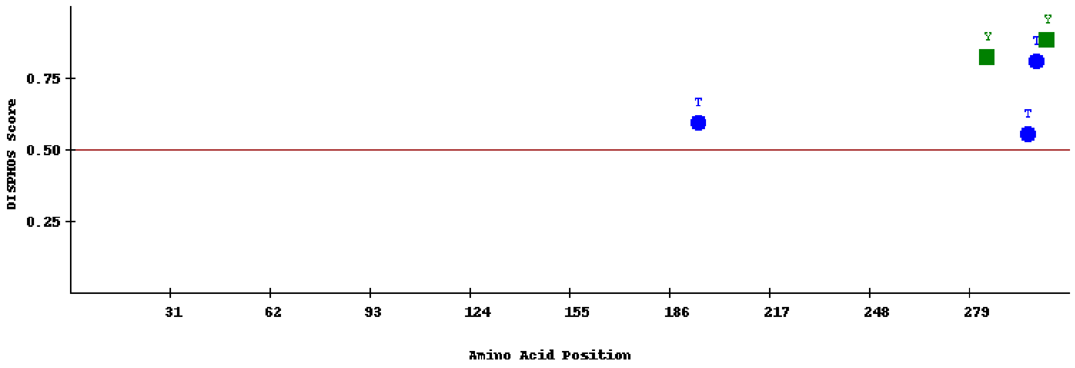


**Supplementary Figure 3.** The phosphorylation modification sites of *Cg*CD63H predicted by DISPHOS 1.3. Sites with score > 0.5 were deemed as positive signals.

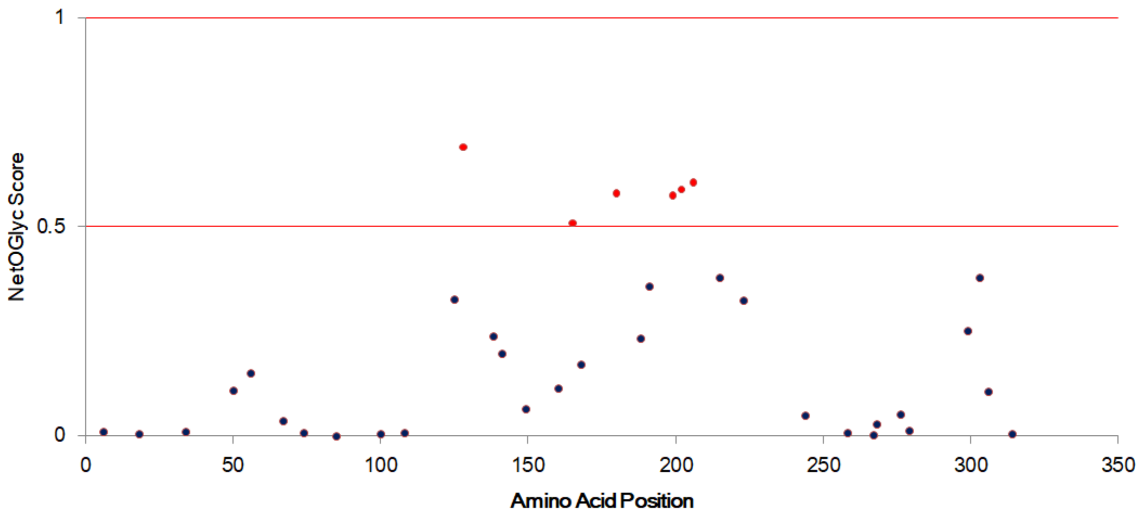


**Supplementary Figure 4.** The O-linked glycosylation modification sites of *Cg*CD63H predicted by NetOGlyc 4.0 Server. Sites with score > 0.5 were deemed as positive signals.

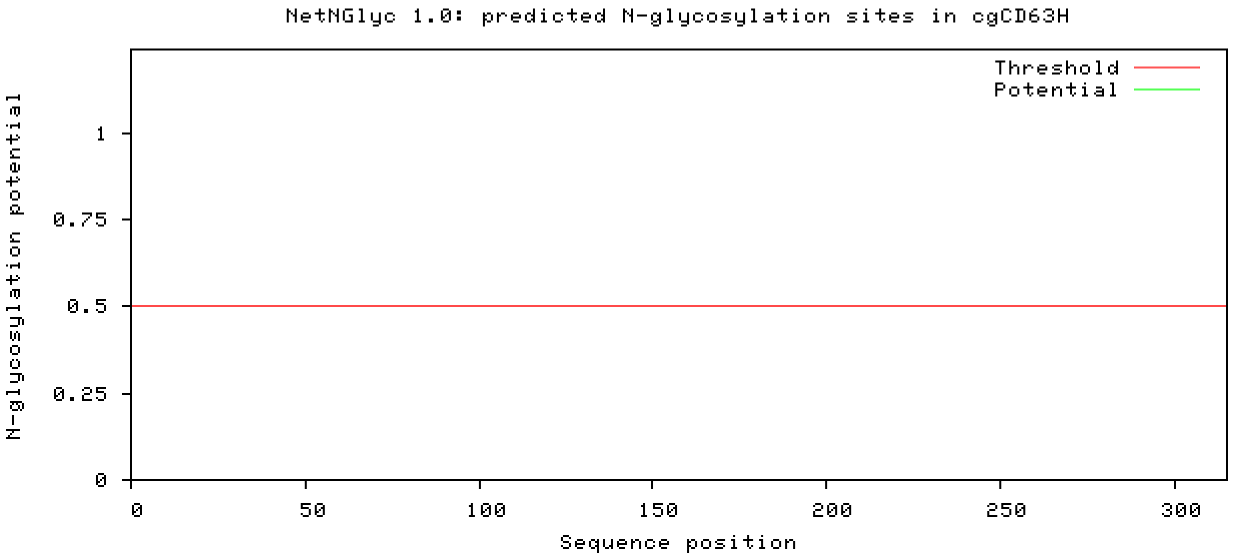


**Supplementary Figure 5.** The N-linked glycosylation modification sites of *Cg*CD63H predicted by NetNGlyc 1.0 Server. No positive signal was found with score > 0.5.

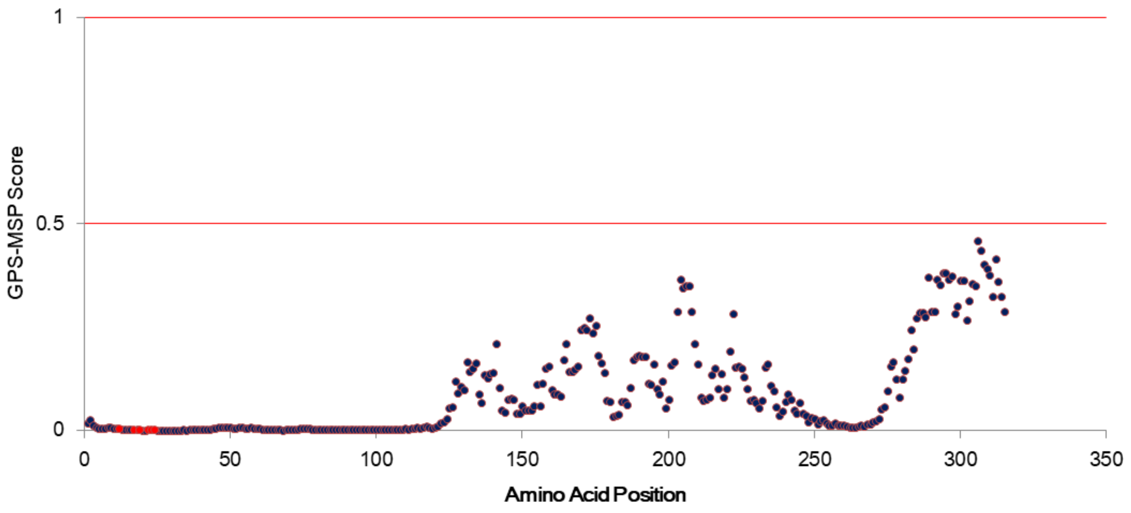


**Supplementary Figure 6.** The methylation modification sites of *Cg*CD63H predicted by GPS-MSP Online Service. No positive signal was found with score > 0.5.

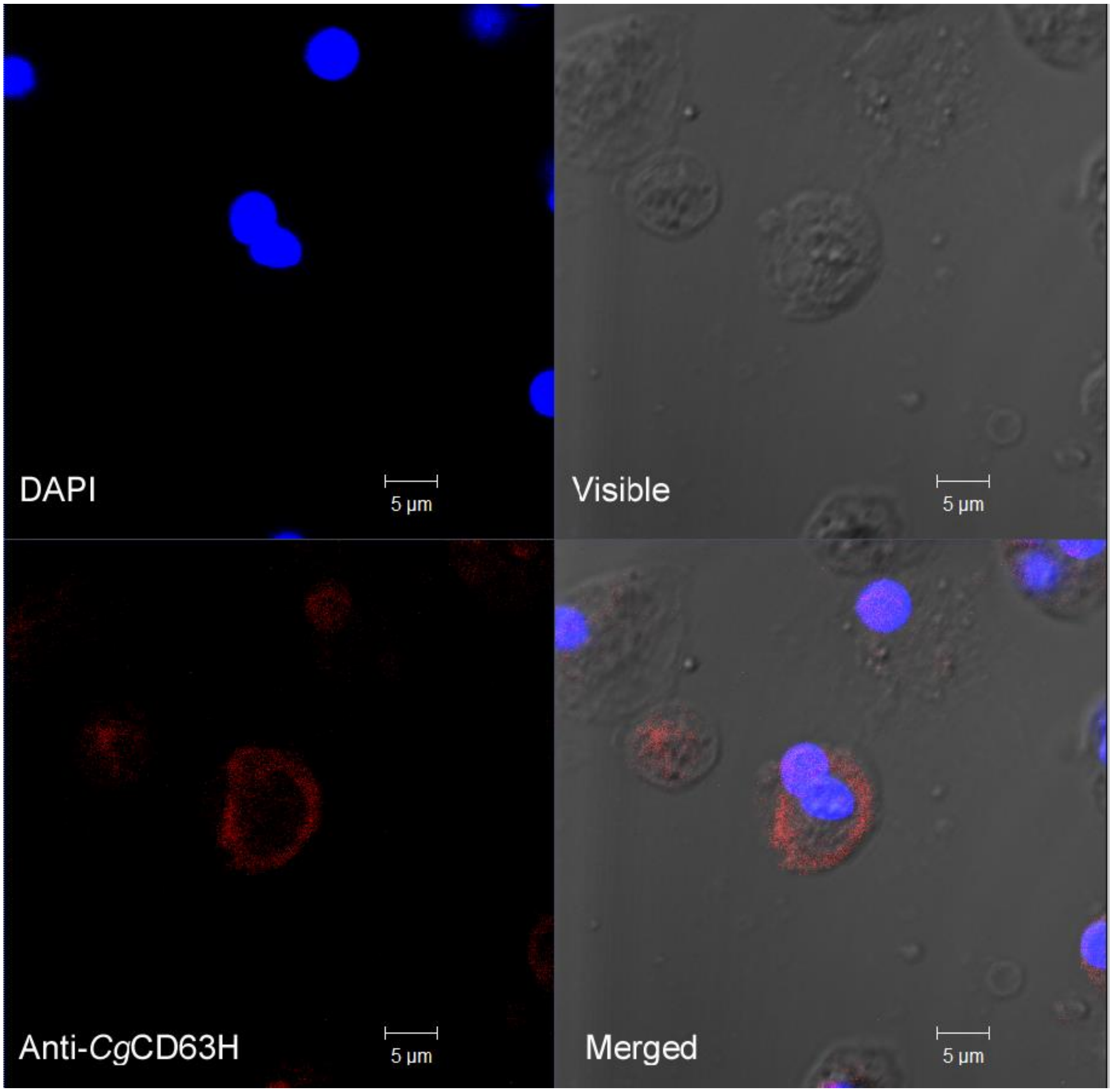


**Supplementary Figure 7.** Subcellular localization of CgCD63H protein in oyster hemocytes (positive  and control). Binding of antibody to *Cg*CD63H was visualized by DyLight 594-labeled secondary antibody (red), the nucleus of hemocytes was stained with DAPI (blue), bar = 5 μm.

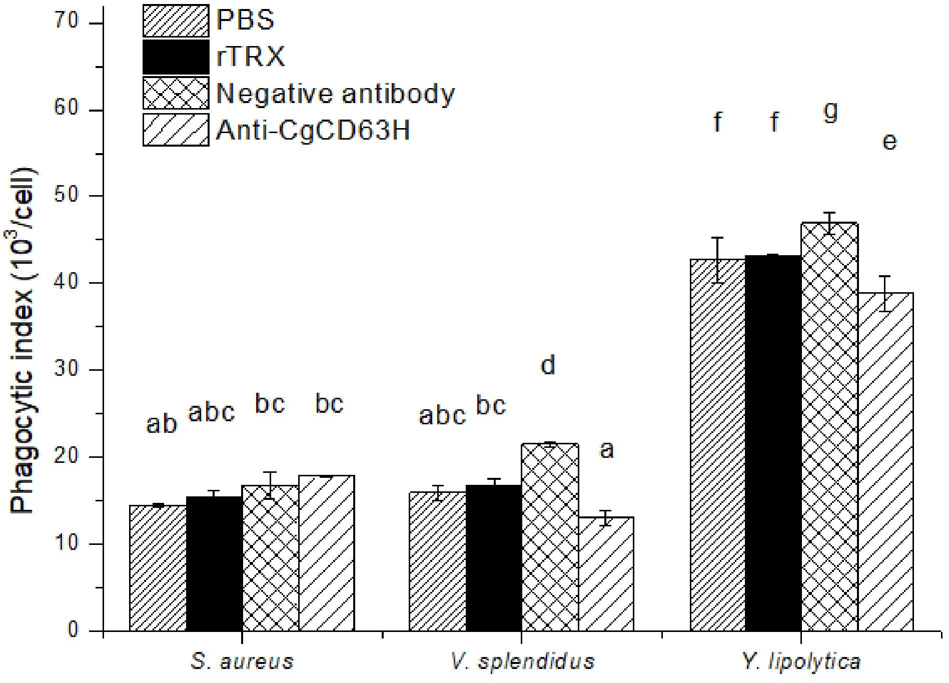


**Supplementary Figure 8.** Hemocyte phagocytosis index (mean FITC fluorescent intensity) post the incubation of anti-CgCD63H detected by flow cytometry. Oyster hemocytes were employed to analyze the change of hemocytes phagocytosis rate against *S. aureus*, *V. splendidus* and *Y. lipolytica* post the incubation of anti-CgCD63H. FITC labeled microbe, anti-CgCD63H and hemocytes were mixed and incubated for 1 h in dark. Recombinant protein rTRX and negative antibody were employed as negative controls. For each treatment, assay was performed in three different replicates for statistical analysis ($p < 0.01$).
